# Antimicrobial susceptibility and risk factors for resistance among *Escherichia coli* isolated from canine specimens submitted to a diagnostic laboratory in Indiana, 2010–2019

**DOI:** 10.1371/journal.pone.0263949

**Published:** 2022-08-24

**Authors:** John E. Ekakoro, G. Kenitra Hendrix, Lynn F. Guptill, Audrey Ruple

**Affiliations:** 1 Department of Public and Ecosystem Health, College of Veterinary Medicine, Cornell University, Ithaca, NY, United States of America; 2 Department of Comparative Pathobiology, College of Veterinary Medicine, Purdue University, West Lafayette, IN, United States of America; 3 Department of Veterinary Clinical Sciences, College of Veterinary Medicine, Purdue University, West Lafayette, IN, United States of America; 4 Department of Population Health Sciences, Virginia-Maryland College of Veterinary Medicine, Virginia Tech, Blacksburg, VA, United States of America; Nitte University, INDIA

## Abstract

*Escherichia coli* (*E*. *coli*) is the most common Gram-negative pathogen isolated in human infections. Antimicrobial resistant (AMR) *E*. *coli* originating from dogs may directly or indirectly cause disease in humans. The objective of this study was to calculate the proportion of antimicrobial susceptible *E*. *coli* isolated from canine specimens submitted to the Indiana Animal Disease Diagnostic Laboratory and to identify temporal patterns of susceptibility among these isolates. Susceptibility data of 2,738 *E*. *coli* isolates from dogs from 2010 through 2019 were used in this study. Proportions of isolates susceptible to the various antimicrobials were calculated using SAS statistical software and the Cochran-Armitage trend test was used to investigate the temporal trends in susceptibility. A multivariable binary logistic regression model was built to investigate the association between host factors and AMR. Overall, 553/2,738 (20.2%) of the isolates were susceptible to 17 of the 27 antimicrobials examined. Of the 2,638 isolates examined for amikacin susceptibility, 2,706 (97.5%) were susceptible, 2,657/2,673 (99.4%) isolates were susceptible to imipenem, and 2,099/2,670 (78.6%) were susceptible to marbofloxacin. A significant decreasing trend in susceptibility was observed for amoxicillin-clavulanic acid (*P*<0.0001), ampicillin (*P*<0.0001), Cefazolin (*P*<0.0001), ceftazidime (*P* = 0.0067), chloramphenicol (*P*<0.0001), and orbifloxacin (*P* = 0.008). The overall percentage of AMR isolates (isolates not susceptible to at least one antimicrobial) was 61.7% (1,690/2,738) and 29.3% (801/2,738) of isolates were multidrug resistant. Multivariable regression analyses showed significant associations between AMR and age (*P* = 0.0091), breed (*P* = 0.0008), and sample isolation site/source (*P*<0.0001). The decreasing trend in the proportion of isolates susceptible to several beta-lactam antimicrobials suggests that resistance of *Escherichia coli* in dogs to these antimicrobials could be increasing in Indiana. The decreasing trend in susceptibility to these drugs could be due to selection pressure from antimicrobial use.

## Introduction

*Escherichia coli*, a member of the ESBL-producing Enterobacteriaceae, is the most common Gram-negative pathogen isolated in human clinical infections, and antimicrobial resistant (AMR) *E*. *coli* pose a threat to both human and animal health [1]. Previous studies have reported isolation of transmissible AMR *E*. *coli* in dogs [2]. *E*. *coli* is the most common cause of urinary tract infections in humans and dogs and sharing of *E*. *coli* strains between dogs and humans can occur [3]. The CDC reported that an estimated 197,400 cases of and 9,100 deaths occurred due to ESBL-Enterobacteriaceae infections among hospitalized patients in 2017 in the US [4]. AMR *E*. *coli* originating from dogs may directly or indirectly cause disease in humans [5].

However, we do not know the total number of cases in which AMR *E*. *coli* cause disease or death in dogs in the US. Without this knowledge, we cannot fully understand the role dogs may play in spreading AMR *E*. *coli* infections to humans. In addition, understanding the patterns of antimicrobial susceptibility of bacterial isolates identified from dogs is a critical step in antimicrobial stewardship and in the containment of AMR within the One Health framework. The objectives of this study were to: 1) calculate the proportion of antimicrobial susceptible E. coli isolates identified in canine specimens submitted to the Indiana Animal Disease Diagnostic Laboratory (ADDL) from January 1, 2010, through December 1, 2019; 2) identify temporal trends in susceptibility among these isolates to individual antimicrobials tested; and 3) to identify the temporal patterns and host risk factors for AMR and multidrug resistance (MDR) among these isolates.

## Materials and methods

### Source of data and ethical approval

The study was exempted from oversight by the Purdue University Institutional Animal Care and Use Committee (IACUC). We used secondary data obtained from the Indiana ADDL and informed consent was not required. No field studies or experiments were conducted in this study, and the study did not directly involve use of animals and posed no risk to clients (animal owners). Data from *E*. *coli* isolates phenotypically assessed for AMR from January 1, 2010, through December 31, 2019, were utilized. The variables extracted from the dataset included: the age of the dog, breed, sex, geographic location (localized to zip code) of its home, and host source (anatomic location) of isolation of the pathogen.

The antimicrobial susceptibility test (AST) results used in this analysis were obtained using the broth microdilution method using the Sensititre™ Companion Animal Gram Negative COMPGN1F Vet AST Plates purchased from ThermoFisher scientific-USA, the Mueller-Hinton broth as the media, and *Escherichia coli* (ATCC® 25922™) as the quality control strain. All testing was in accordance with the ADDL standard operating procedure for broth microdilution method. This yielded quantitative data (minimum inhibitory concentration) and the isolates were categorized as susceptible (S), intermediate (I), or resistant (R) based upon Clinical and Laboratory Standards Institute (CLSI) guidelines that were current at the time the isolate was tested [6]. The susceptibility testing was performed for 35 drugs: amikacin, amoxicillin, ampicillin, azithromycin, cefazolin, cefovecin, cefoxitin, cefpodoxime, ceftazidime, ceftiofur, chloramphenicol, chlortetracycline, clarithromycin, clindamycin, danofloxacin, doxycycline, enrofloxacin, erythromycin, florfenicol, gentamicin, imipenem, marbofloxacin, neomycin, oxacillin, oxytetracycline, penicillin, rifampin, spectinomycin, sulfadimethoxine, tetracycline, tiamulin, ticarcillin, ticarcillin-clavulanate, tilmicosin, trimethoprim, tulathromycin, and tylosin. Drugs with complete susceptibility data or with more than 500 isolates tested were considered in these analyses.

Overall, 27 antimicrobials from 10 antimicrobial classes were included in the final analyses. The antimicrobial classification conformed with the classification described by Riviere and Papich [7] and the 10 classes included aminoglycosides, the penicillins, cephalosporins and cephamycins, carbapenems, amphenicols, fluoroquinolones, macrolides, lincosamides, tetracyclines, and antifolate. All 10 classes belonged to either critically important antimicrobial classes for human medicine (e.g. aminoglycosides, carbapenems, penicillins) or highly important antimicrobials (e.g. amphenicols, antifolate) as classified by the World Health Organization (WHO) [8]. For AMR and MDR determination, drugs known to exhibit intrinsic resistance phenotypes in Enterobacteriaceae [9] (e.g. penicillin, oxacillin, clindamycin, and erythromycin) were excluded.

### Data and statistical analysis

Data cleaning and preparation was performed in Microsoft Excel. The data were assessed for completeness, duplicates were removed, and only complete records were included in the analyses. Geographic origins of the samples located to zip code were categorized at the county and state spatial scales. The state spatial scale categories were further grouped into within Indiana, out-of-state, and unknown (for those where no geographic origin was reported). The sex of the dog was categorized as male, female, or intersex regardless of neuter status. Age was categorized into seven age groups: less than 1 year, 1 to 3 years, over 3 to 6 years, over 6 to 8 years, over 8 to 10years, over 10 to 12years, and greater than 12 years of age as described previously [10]. We removed one case from the age category due to an implausible age designation of 95 years.

Dog breeds were grouped based on the American Kennel Club (AKC) breed group classification as described by Conner and colleagues [11]. However, three breeds (English shepherd, Jack Russel terrier, and Pitbull) that were not listed on the AKC grouping system were classified based on the United Kennel Club (UKC) grouping [12]. Dogs identified in the dataset as mixed breed were treated as such in the final grouping. Two breeds (goldendoodle and cockapoo) that were not yet recognized by any major kennel club were included in the category mixed. If an animal was identified using a non-specific breed name such as poodle, or schnauzer, they were categorized as unknown breed. If breed, sex, or age of the dog was not reported and other data was otherwise complete, it was categorized as “unknown” for the specific category.

The anatomic location or specimen source was categorized as: abdominal cavity/fluid, ear and ocular, feces, respiratory tract, skin, urine and bladder, uterus, vagina and vulva, wounds, and “all others.” The “all others” contained specimen sources with very small counts or those with non-specific identities such as fluid, swabs, tissue etc. All AST results reported as “NI” (no interpretation) were excluded from the analysis. A more conservative approach for categorization of all AST data reported as susceptible, intermediate, or resistant was adopted for this study as previously suggested by Sweeney and others [13] and Magiorakos and others [14]. Briefly, the AST data were grouped into two categories “susceptible” and “not susceptible.” The “not susceptible” category included the resistant and/or intermediately susceptible isolates. Isolates that were not susceptible to at least one antimicrobial drug were considered to be AMR isolates [11] and isolates that were not susceptible to at least one antimicrobial drug in at least three antimicrobial classes were considered to be MDR as previously described [13]. The CLSI guidelines were used in the analysis of the AST results [15].

#### Descriptive analyses

Statistical analyses were performed in a SAS commercial statistical software. Frequencies and proportions were used to summarize the data. The Cochran-Armitage trend test was used to investigate the temporal trends in the data.

#### Univariable and multivariable analysis

Isolates from intersex dogs and from dogs belonging to the foundation stock service breed group were excluded from the univariable and multivariable analyses due to small counts. Univariable binary logistic regression was used to investigate the association between geographic origin of sample and AMR. A further analysis of the associations between host factors (age, sex, and breed of the dog, specimen source/type and AMR/MDR) were conducted only for samples with a known in-state address. Variables with a *p*-value ≤ 0.15 in the univariable analysis were considered for inclusion in the multivariable model building. A multivariable binary logistic regression model was built to investigate the association between host factors and AMR. The backward elimination procedure was used to build the multivariable model and only statistically significant predictors (*P*≤ 0.05) were retained in the final main effects multivariable model. In the final model, two-way interactions between age and breed were assessed based on biological plausibility and standard multiple pairwise comparisons were obtained using the SAS “LSMEANS” statement. The model fit was assessed using The Hosmer and Lemeshow Goodness-of-Fit Test. Cluster analysis to discern the spatial patterns of AMR/MDR was deemed untenable due to small sample sizes in the different counties in Indiana.

## Results

### Sample characteristics

A total of 2,738 *E*. *coli* isolates were included in the general analysis of these data. Of these, 1,641 (59.9%) were isolated from samples obtained from female dogs, 881 (32.2%) from male dogs, three (0.1%) were from intersex dogs, and 190 (7%) samples were from dogs that did not have sex identified. Most of the samples (n = 2,058; 75.2%) were identified using an in-state zip code while 275 (10%) were identified as being from out-of-state samples; 405 (14.8%) samples had no geographic origin reported. Out-of-state samples came from 18 states: Illinois (n = 175), Michigan (n = 23), Ohio (n = 23), Maryland (n = 10), Tennessee (n = 9), Missouri (n = 5), Georgia (n = 5), West Virginia (n = 5), California (n = 4), Kentucky (n = 4), Florida (n = 3), Texas (n = 2), Pennsylvania (n = 2), Virginia (n = 1), Wisconsin (n = 1), Nebraska (n = 1), Alabama (n = 1), and Arkansas (n = 1) (Table 1).

**Table 1:** Characteristics of all *Escherichia coli* isolates tested for antimicrobial susceptibility at the Indiana Animal Disease Diagnostic Laboratory, from January 2010 to December 2019.

Sample characteristics	Number (%) of isolates
**Geographic origin of sample**	**N = 2,738**
Indiana	2,058 (75.2)
Out-of-state	275 (10)
Location not recorded	405 (14.8)
**Sex**	**N = 2,738**
Female	1,641 (59.9)
Male	881 (32.2)
Intersex	3 (0.1)
Unknown	213 (7.8)
**Age of dog (years)**	**N = 2,737**
<1year	208 (7.6)
1-3years	265 (9.7)
>3-6years	440 (16.1)
>6-8years	413 (15.1)
>8-10years	496 (18.1)
>10-12years	447 (16.3)
>12years	408 (14.9)
Unknown	60 (2.2)
**Breed Group**	**N = 2,738**
Mixed breed	583 (21.3)
Sporting	565 (20.6)
Working	312 (11.4)
Hound	256 (9.4)
Terrier	256 (9.4)
Toy	252 (9.2)
Herding	222 (8.1)
Non-Sporting	200 (7.3)
Unknown	88 (3.2)
Foundation Stock Service	4 (0.2)
**Isolation source**	**N = 2,738**
Abdominal cavity and fluid	77 (2.8)
Ear and Ocular	138 (5)
Feces	170 (6.2)
Respiratory tract	101 (3.7)
Skin	45 (1.6)
Urine and bladder	1676 (61.2)
Uterus, vagina, and vulva	59 (2.2)
Wounds	71 (2.6)
All others	401 (14.7)
**Year of sample collection**	**N = 2,738**
2010	206 (7.5)
2011	249 (9.1)
2012	228 (8.3)
2013	232 (8.5)
2014	280 (10.2)
2015	257 (9.4)
2016	310 (11.3)
2017	294 (10.7)
2018	355 (13)
2019	327 (12)

### Proportions and trends in susceptibility to different antimicrobials

Overall, 553 (20.2%) of the isolates were susceptible to 17 of the 27 antimicrobials examined. *E*. *coli* susceptibility to marbofloxacin was 78.6% (2,099/2,670) and ranged from 83.3% (170/204) susceptible isolates tested in 2010 to 75.7% (234/309) susceptible isolates tested in 2019. Overall susceptibility to doxycycline was 74.4% (1,999/2,688) and ranged from 77.5% (158/204) susceptible isolates tested in 2010 to 72.5 (227/313) susceptible isolates tested in 2019 (Table 2). Statistically significant temporal trends were observed among 10 of the 27 antimicrobials evaluated (Table 2). A significant (*P* < 0.05) downward (decreasing) trend in susceptibility was observed for amoxicillin-clavulanic acid, ampicillin, cefalexin, cefazolin, ceftazidime, cephalothin, chloramphenicol, and orbifloxacin (Table 2).

**Table 2:** Trends in antimicrobial susceptibility of *Escherichia coli* isolated from dog specimens tested at the Indiana Animal Disease Diagnostic Laboratory, 2010–2019.

Antimicrobial class	Antimicrobial	Percentage (number of specimens tested) of susceptible isolates to an antimicrobial										Total	Statistic (Z)- CAT-T	P-values (CAT-T)
		2010	2011	2012	2013	2014	2015	2016	2017	2018	2019			
Aminoglycosides														
	Amikacin	97.6 (204)	98.8 (248)	95.6 (226)	96.1 (232)	93.9 (277)	98.1 (257)	97.1 (310)	100 (289)	99.2 (354)	97.3 (309)	97.5 (2706)	-2.1528	0.0157
	Gentamycin	86.4 (206)	93.6 (249)	84.7 (228)	83.2 (232)	87.9 (280)	90.3 (257)	89.4 (310)	92.9 (294)	84.2 (355)	89.3 (327)	88.2 (2738)	-0.3426	0.3660
Amphenicols	Chloramphenicol	89.2 (203)	91.1 (248)	83.2 (226)	80.6 (232)	86.3 (277)	80.2 (257)	83.9 (310)	82.7 (289)	75.5 (351)	78.8 (217)	82.8 (2610)	4.8084	< .0001
Antifolate	Trimethoprim	82 (206)	86.8 (249)	75.4 (228)	75.9 (232	76.8 (280)	81.3 (257)	81.9 (310)	83.6 (293)	74.7 (348)	78.5 (311)	79.6 (2714)	1.2911	0.0983
Carbapenem	Imipenem	99 (204)	100 (248)	99.1 (226)	99.6 (230)	98.9 (275)	100 (256)	99.7 (306)	99.3 (283)	99.7 (336)	98.7 (309)	99.4 (2673)	0.4271	0.3346
Cefalosporin/Cefamycin														
	Cefalexin	-	-	-	-	-	-	63.5 (63)	78.3 (281)	61.5 (327)	66 (300)	67.9 (971)	2.1955	0.0141
	Cefazolin	74.3 (202)	75.8 (248)	73 (226)	68.5 (232)	75.1 (277)	73.5 (257)	59.1 (308)	69 (284)	54.6 (339)	51.4 (313)	66.4 (2686)	8.1388	< .0001
	Cefovecin	75 (204)	77 (248)	72.1 (226)	69.6 (230)	78.2 (275)	75.8 (256)	72.9 (306)	84.4 (282)	67.4 (331)	68.9 (309)	74 (2667)	1.4236	0.0773
	Cefoxitin	76.5 (204)	79.8 (248)	74.3 (226)	72.6 (230)	80.7 (275)	82.8 (256)	77 (243)	0	0 (1)	0	77.84 (1683)	-0.8763	0.1904
	Cefpodoxime	74 (204)	76.2 (248)	71.7 (226)	71.3 (230)	77.8 (275)	75.4 (256)	71.9 (306)	84.1 (283)	66.7 (336)	67.6 (309)	73.5 (2673)	1.6614	0.0483
	Ceftazidime	-	-	-	-	-	-	85.7 (63)	89.7 (281)	82.3 (327)	81.3 (300)	84.4 (971)	2.4729	0.0067
	Ceftiofur	75.2 (206)	74.3 (249)	71.5 (228)	66.4 (232)	75.7 (280)	73.5 (257)	73.3 (247)	72.7 (11)	85.7 (21)	79 (19)	73.1 (1750)	-0.3796	0.3521
	Cephalothin	-	76.5 (115)	60.2 (226)	51.1 (141)	-	-	-	0 (2)	0 (9)	7.7 (13)	58.7 (506)	6.7500	< .0001
Penicillins														
	Amoxiclav	72.6 (204)	67.2 (137)	100 (2)	71.4 (91)	69.5 (275)	76.2 (256)	65.6 (299)	48.1 (283)	46.4 (336)	44.4 (288)	60.3 (2171)	9.3130	< .0001
	Ampicillin	59.2 (206)	55.4 (139)	50 (2)	55.3 (94)	53.6 (278)	57.8 (256)	50.7 (306)	37.2 (288)	37.1 (337)	38.2 (275)	47.7 (2183)	7.1012	< .0001
	Penicillin	0 (206)	0 (247)	0 (228)	0 (229)	0 (276)	0 (256)	0 (243)	0 (7)	0 (12)	0 913)	0 (1717)	-	-
	Oxacillin	0.5 (204)	0.8 (248)	2.2 (226)	1.3 (230)	1.5 (275)	0 (256)	1.7 (243)	0 (2)	0 (10)	7.7 (13)	1.2 (1707)	-0.6857	0.2465
	Piperacillin tazobactam	-	-	-	-	-	-	100 (63)	96.4 (281)	97 (326)	97.3 (300)	97.1 (970)	0.2269	0.4103
	Ticarcillin	60.8 (204)	58.1 (248)	54.4 (226)	52.2 (232)	54.9 (277)	58.4 (257)	63.2 (247)	83.3 (6)	52.6 (19)	72.2 (18)	57.6 (1734)	-0.9315	0.1758
	Ticarcillin Clav	72.6 (204)	70.2 (248)	70.8 (226)	64.4 (230)	65.5 (275)	70.3 (256)	67.9 (243)	0	0 (1)	0	68.6 (1683)	1.2077	0.1136
Fluoroquinolones														
	Enrofloxacin	83 (206)	80.3 (249)	74.1 (228)	73 (230)	79.5 (278)	78.9 (256)	76.8 (306)	91.7 (266)	73.3 (326)	73.1 (309)	78.2 (2654)	1.0780	0.1405
	Marbofloxacin	83.3 (204)	81.9 (248)	74.3 (226)	74.4 (230)	80.4 (275)	78.9 (256)	77.5 (306)	88.3 (282)	73.1 (334)	75.7 (309)	78.6 (2670)	1.2731	0.1015
	Orbifloxacin	-	-	-	-	-	-	71.4 (63)	85.4 (280)	72.3 (325)	73 (300)	76.2 (968)	2.3941	0.0083
Lincosamide	Clindamycin	0 (206)	0 (249)	0 (228)	0 (230)	0 (278)	0 (256)	0 (243)	0 (7)	0 (12)	0 (14)	0.06 (1723)	-2.7964	0.0026
Macrolide	Erythromycin	0 (204)	0 (248)	0 (226)	0 (232)	0 (277)	0 (257)	0 (202)	-	-	-	0 (1646)	-	-
Tetracyclines														
	Doxycycline	77.5 (204)	76.6 (248)	72.1 (226)	68.4 (231)	75.8 (277)	73.4 (256)	79.2 (307)	75.9 (286)	72.1 (340)	72.5 (313)	74.4 (2688)	0.5936	0.2764
	Tetracycline	-	-	-	-	-	-	81 (63)	74.4 (285)	70 (327)	72.1 (301)	72.6 (976)	1.3344	0.0910

### Antimicrobial resistance (AMR) and multi-drug resistance (MDR)

**Fig 1:**
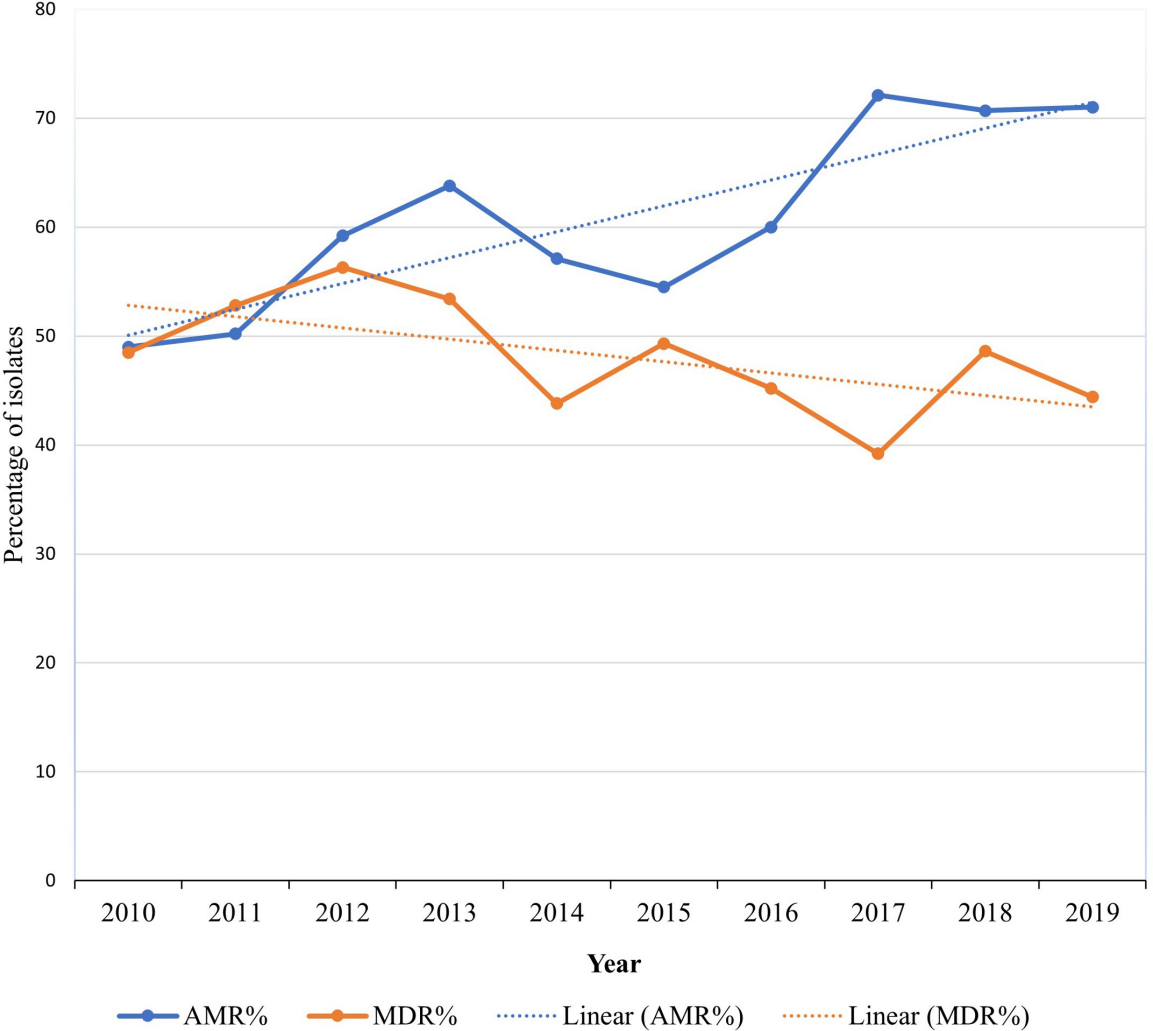
A graphical representation of the temporal trends in antimicrobial resistance and multidrug resistance among *Escherichia coli* isolated from dog specimens at the Indiana Animal Disease Diagnostic Laboratory, 2010–2019.

The overall percentage of AMR (isolates not susceptible to at least one antimicrobial) in isolates was 61.7% (n = 1,690) and 29.3% (801) of isolates were MDR. Of the 1,690 AMR isolates, 47.4% (801/1,690) were MDR (Table 3). A significant (*P* = <0.0001) upward trend in AMR was observed while MDR significantly (*P* = 0.0083) decreased (Fig 1). Geographic region of sample origin (e.g., out-of-state versus in-state) was significantly associated with AMR (*P* < .0001). The odds of an isolate being shown to have resistance to at least one antimicrobial were two times higher in all (combined) out-of-state samples when compared to samples from Indiana (OR: 2.04, 95% CI: 1.54–2.7) and the odds of an isolate being shown to have resistance to at least one antimicrobial were 1.89 times higher among samples of unreported (unknown) origin when compared to known Indiana samples (OR: 1.89, 95% CI:1.5–2.39).

**Table 3:** Trends in antimicrobial resistance and multidrug resistance among *Escherichia coli* isolated from dog specimens at the Indiana Animal Disease Diagnostic Laboratory, 2010–2019.

	Percentage (number of specimen tested) of AMR/MDR isolates										Total	Statistic (Z)- CAT-T	*P*-values (CAT-T)
	2010	2011	2012	2013	2014	2015	2016	2017	2018	2019			
AMR	49 (206)	50.2 (249)	59.2 (228)	63.8 (232)	57.1 (280)	54.5 (257)	60 (310)	72.1 (294)	70.7 (355)	71 (327)	61.7 (2738)	-7.4123	< .0001
MDR	48.5 (101)	52.8 (125)	56.3 (135)	53.4 (148)	43.8 (160)	49.3 (140)	45.2 (186)	39.2 (212)	48.6 (251)	44.4 (232)	47.4 (1690)	2.3959	0.0083

#### Host factors associated with AMR/MDR in Indiana

For all samples from known Indiana addresses, 1,191/2,050 (58.1%) were resistant to at least one antimicrobial and 859/2,050 (41.9%) were not resistant to any antimicrobials. Of the 1,191 AMR isolates, 532 (44.7%) were MDR (Table 4).

**Table 4:** The distribution of isolates from Indiana based on host factors and AMR status.

Host factors	Total number (%) of isolates assessed for AMR	Number (%) of AMR isolates		Total number (%) of isolates assessed for MDR	Number (%) of MDR isolates	
		No	Yes		No	Yes
**Sex**	**N = 2050**			**N = 1191**		
Female	1239 (60.4)	509 (24.8)	730 (35.6)	730 (61.3)	394 (33.1)	336 (28.2)
Male	617 (30.1)	265 (12.9)	352 (17.2)	352 (29.6)	200 (16.8)	152 (12.8)
Unknown	194 (9.5)	85 (4.2)	109 (5.3)	109 (9.2)	65 (5.5)	44 (3.7)
**Age (years)**	**N = 2050**			**N = 1191**		
<1year	177 (8.6)	78 (3.8)	99 (4.8)	99 (8.3)	50 (4.2)	49 (4.1)
1-3years	209 (10.2)	71 (3.5)	138 (6.7)	138 (11.6)	71 (6)	67 (5.6)
>3-6years	319 (15.6)	137 (6.7)	182 (8.9)	182 (15.3)	105 (8.8)	77 (6.5)
>6-8years	330 (16.1)	161 (7.9)	169 (8.2)	169 (14.2)	95 (8)	74 (6.2)
>8-10years	376 (18.3)	166 (8.1)	210 (10.2)	210 (17.6)	132 (11)	78 (6.6)
>10-12years	310 (15.1)	112 (5.5)	198 (9.7)	198 (16.6)	108 (9)	90 (7.6)
>12years	279 (13.6)	114 (5.6)	165 (8)	165 (14)	82 (7)	83 (7)
Unknown	50 (2.4)	20 (0.9)	30 (1.5)	30 (2.5)	16 (1.3)	14 (1.2)
**Breed Group**	**N = 2050**			**N = 1191**		
Sporting	457 (22.3)	206 (10.1)	251 (12.2)	251 (21.1)	145 (12.2)	106 (8.9)
Mixed breed	411 (20.1)	178 (8.7)	233 (11.4)	233 (19.6)	128 (10.8)	105 (8.8)
Working	225 (11)	100 (4.9)	125 (6.1)	125 (10.5)	68 (5.7)	57 (4.8)
Toy	195 (9.5)	88 (4.3)	107 (5.2)	107 (9)	67 (5.6)	40 (3.4)
Hound	184 (9)	85 (4.2)	99 (4.8)	99 (8.3)	58 (4.9)	41 (3.4)
Terrier	182 (8.9)	53 (2.6)	129 (6.3)	129 (10.8)	69 (5.8)	60 (5)
Herding	170 (8.3)	56 (2.7)	114 (5.6)	114 (9.6)	50 (4.2)	64 (5.4)
Non-Sporting	147 (7.2)	53 (2.6)	94 (4.6)	94 (7.9)	52 (4.4)	42 (3.5)
Unknown	79 (3.9)	40 (2)	39 (1.9)	39 (3.3)	22 (1.9)	17 (1.4)
**Isolation source**	**N = 2050**			**N = 1191**		
Abdominal cavity and fluid	65 (3.2)	26 (1.3)	39 (1.9)	39 (3.3)	26 (2.2)	13 (1.1)
Ear and Ocular	112 (5.5)	42 (2.1)	70 (3.4)	70 (5.9)	43 (3.6)	27 (2.3)
Feces	96 (4.7)	32 (1.6)	64 (3.1)	64 (5.4)	36 (3)	28 (2.4)
Respiratory tract	80 (3.9)	17 (0.8)	63 (3.1)	63 (5.3)	27 (2.3)	36 (3)
Skin	30 (1.4)	9 (0.4)	21 (1)	21 (1.8)	13 (1.1)	8 (0.7)
Urine and bladder	1257 (61.3)	584 (28.5)	673 (32.8)	673 (56.5)	374 (31.4)	299 (25.1)
Uterus, vagina, and vulva	43 (2.1)	23 (1.1)	20 (1)	20 (1.7)	14 (1.2)	6 (0.5)
Wounds	52 (2.5)	13 (0.6)	39 (1.9)	39 (3.3)	17 (1.4)	22 (1.9)
All others	315 (15.4)	113 (5.5)	202 (9.9)	202 (17)	109 (9.2)	93 (7.8)

#### Univariable logistic regression

There was no significant unadjusted association between sex and the outcome of AMR, however breed, age, and isolation source had significant associations with AMR (Table 5). There were no significant unadjusted associations between the four host factors and MDR (Table 6).

**Table 5:** Results of univariable logistic regression models assessing the association of host factors with antimicrobial resistance among *Escherichia coli* isolated from dog specimens originating from Indiana.

Host factors	Category	OR (95%CI)	*P* Value
Sex	^†^Overall	─	0.6338
	Male vs Female	0.93 (0.76–1.13)	0.442
	Male vs Unknown	1.04 (0.75–1.43)	0.832
	Female vs Unknown	1.12 (0.82–1.52)	0.473
Age	^†^Overall	─	0.0149
	1-3years vs >3-6years	1.46 (1.02–2.1)	0.039
	1-3years vs >6-8years	1.85 (1.29–2.65)	0.0008
	1-3years vs >8-10years	1.54 (1.08–2.18)	0.017
	1-3years vs >10-12years	1.1 (0.76–1.59)	0.614
	1-3years vs >12years	1.34 (0.93–1.95)	0.121
	1-3years vs Unknown	1.3 (0.69–2.44)	0.423
	1-3years vs <1year	1.53 (1.01–2.31)	0.043
	>3-6years vs >6-8years	1.27 (0.93–1.73)	0.136
	>3-6years vs >8-10years	1.05 (0.78–1.42)	0.750
	>3-6years vs >10-12years	0.75 (0.55–1.04)	0.081
	>3-6years vs >12years	0.92 (0.66–1.27)	0.606
	>3-6years vs Unknown	0.89 (0.48–1.63)	0.695
	>3-6years vs <1year	1.05 (0.72–1.52)	0.809
	>6-8years vs >8-10years	0.83 (0.62–1.12)	0.218
	>6-8years vs >10-12years	0.59 (0.43–0.82)	0.001
	>6-8years vs >12years	0.73 (0.53–1)	0.05
	>6-8years vs Unknown	0.7 (0.38–1.28)	0.248
	>6-8years vs <1year	0.83 (0.57–1.19)	0.310
	>8-10years vs >10-12years	0.72 (0.53–0.94)	0.034
	>8-10years vs >12years	0.87 (0.64–1.2)	0.4
	>8-10years vs Unknown	0.84 (0.46–1.54)	0.579
	>8-10years vs <1year	1 (0.7–1.43)	0.986
	>10-12years vs >12years	1.22 (0.88–1.7)	0.239
	>10-12years vs Unknown	1.18 (0.64–2.17)	0.598
	>10-12years vs <1year	1.39 (0.96–2.03)	0.085
	>12years vs Unknown	0.97 (0.52–1.78)	0.909
	>12years vs <1year	1.14 (0.78–1.67)	0.499
	Unknown vs <1year	1.18 (0.62–2.24)	0.608
Breed group	^†^Overall	─	0.0007
	Hound vs Mixed	0.89 (0.63–1.26)	0.512
	Hound vs non-Sporting	0.66 (0.42–1.02)	0.064
	Hound vs Sporting	0.96 (0.68–1.35)	0.797
	Hound vs Terrier	0.48 (0.31–0.74)	0.0008
	Hound vs Toy	0.96 (0.64–1.44)	0.835
	Hound vs Unknown	1.2 (0.71–2.03)	0.509
	Hound vs Working	0.93 (0.63–1.38)	0.723
	Hound vs Herding	0.57 (0.37–0.88)	0.011
	Mixed vs non-Sporting	0.74 (0.5–1.09)	0.126
	Mixed vs Sporting	1.07 (0.82–1.41)	0.601
	Mixed vs Terrier	0.54 (0.37–0.78)	0.001
	Mixed vs Toy	1.08 (0.76–1.52)	0.673
	Mixed vs Unknown	1.34 (0.83–2.18)	0.231
	Mixed vs Working	1.05 (0.76–1.45)	0.783
	Mixed vs Herding	0.64 (0.44–0.94)	0.021
	Non-Sporting vs Sporting	1.46 (0.99–2.14)	0.055
	Non-Sporting vs Terrier	0.73 (0.46–1.16)	0.182
	Non-Sporting vs Toy	1.46 (0.94–2.26)	0.092
	Non-Sporting vs Unknown	1.82 (1.04–3.17)	0.035
	Non-Sporting vs Working	1.42 (0.93–2.18)	0.109
	Non-Sporting vs Herding	0.87 (0.55–1.39)	0.561
	Sporting vs Terrier	0.5 (0.35–0.72)	0.0002
	Sporting vs Toy	1 (0.72–1.4)	0.99
	Sporting vs Unknown	1.25 (0.78–2.02)	0.361
	Sporting vs Working	0.98 (0.71–1.34)	0.876
	Sporting vs Herding	0.6 (0.41–0.87)	0.006
	Terrier vs Toy	2 (1.31–3.07)	0.001
	Terrier vs Unknown	2.5 (1.45–4.3)	0.001
	Terrier vs Working	1.95 (1.29–2.95)	0.002
	Terrier vs Herding	1.2 (0.76–1.88)	0.439
	Toy vs Unknown	1.25 (0.74–2.11)	0.408
	Toy vs Working	0.97 (0.66–1.43)	0.888
	Toy vs Herding	0.6 (0.39–0.92)	0.018
	Unknown vs Working	0.78 (0.47–1.3)	0.343
	Unknown vs Herding	0.48 (0.28–0.83)	0.008
	Working vs Herding	0.61 (0.41–0.93)	0.02
Sample source/sample type	^†^Overall	─	< .0001
	Ear & ocular vs Feces	0.83 (0.47–1.48)	0.532
	Ear & ocular vs Respiratory tract	0.45 (0.23–0.87)	0.017
	Ear & ocular vs Skin	0.71 (0.3–1.7)	0.448
	Ear & ocular vs Urine & bladder	1.45 (0.97–2.15)	0.069
	Ear & ocular vs Uterus, vagina, vulva	1.92 (0.94–3.9)	0.073
	Ear & ocular vs Wounds	0.56 (0.27–1.16)	0.117
	Ear & ocular vs All others	0.93 (0.6–1.46)	0.758
	Ear & ocular vs Abdominal cavity/fluid	1.1 (0.59–2.08)	0.742
	Feces vs Respiratory tract	0.54 (0.27–1.07)	0.077
	Feces vs Skin	0.86 (0.35–2.09)	0.734
	Feces vs Urine & bladder	1.74 (1.12–2.69)	0.014
	Feces vs Uterus, vagina, vulva	2.3 (1.1–4.79)	0.026
	Feces vs Wounds	0.67 (0.31–1.42)	0.294
	Feces vs All others	1.12 (0.69–1.81)	0.649
	Feces vs Abdominal cavity/fluid	1.3 (0.69–2.56)	0.389
	Respiratory tract vs Skin	1.59 (0.62–4.09)	0.338
	Respiratory tract vs Urine & bladder	3.2 (1.86–5.56)	< .0001
	Respiratory tract vs Uterus, vagina, vulva	4.26 (1.91–9.52)	0.0004
	Respiratory tract vs wounds	1.24 (0.54–2.82)	0.616
	Respiratory tract vs all others	2.07 (1.16–3.71)	0.014
	Respiratory tract vs Abdominal cavity/fluid	2.47 (1.19–5.13)	0.015
	Skin vs Urine & bladder	2.03 (0.92–4.46)	0.08
	Skin vs Uterus, vagina, vulva	2.68 (1–7.18)	0.049
	Skin vs Wounds	0.78 (0.29–2.12)	0.623
	Skin vs All others	1.31 (0.58–2.95)	0.521
	Skin vs Abdominal cavity/fluid	1.56 (0.62–3.92)	0.349
	Urine & bladder vs Uterus, vagina, vulva	1.33 (0.72–2.44)	0.365
	Urine & bladder vs Wounds	0.38 (0.2–0.73)	0.003
	Urine & bladder vs All others	0.65 (0.5–0.83)	0.0008
	Urine & bladder vs Abdominal cavity/fluid	0.77 (0.46–1.28)	0.31
	Uterus, vagina, vulva vs Wounds	0.29 (0.12–0.69)	0.005
	Uterus, vagina, vulva vs All others	0.49 (0.26–0.92)	0.028
	Uterus, vagina, vulva vs Abdominal cavity/fluid	0.58 (0.27–1.26)	0.17
	Wounds vs All others	1.68 (0.86–3.28)	0.129
	Wounds vs Abdominal cavity/fluid	2 (0.9–4.45)	0.09
	All others vs Abdominal cavity/fluid	1.19 (0.69–2.06)	0.53

^†^Overall = overall effect of host factor on AMR.

**Table 6:** Results of univariable logistic regression models assessing the association of host factors with multi-drug resistance among *Escherichia coli* isolated from dog specimens originating from Indiana.

Host factors	Category	OR (95%CI)	*P* Value
Sex	^†^Overall	─	0.4330
	Male vs Female	0.89 (0.69–1.15)	0.378
	Male vs Unknown	1.12 (0.73–1.74)	0.604
	Female vs Unknown	1.26 (0.84–1.9)	0.269
Age	^†^Overall	─	0.2377
	1-3years vs >3-6years	1.29 (0.83–2.01)	0.267
	1-3years vs >6-8years	1.21 (0.77–1.9)	0.405
	1-3years vs >8-10years	1.6 (1.03–2.47)	0.035
	1-3years vs >10-12years	1.13 (0.73–1.75)	0.576
	1-3years vs >12years	0.93 (0.59–1.47)	0.761
	1-3years vs Unknown	1.08 (0.49–2.38)	0.852
	1-3years vs <1year	0.96 (0.58–1.6)	0.886
	>3-6years vs >6-8years	0.94 (0.62–1.44)	0.78
	>3-6years vs >8-10years	1.24 (0.83–1.86)	0.297
	>3-6years vs >10-12years	0.88 (0.59–1.32)	0.537
	>3-6years vs >12years	0.72 (0.47–1.11)	0.136
	>3-6years vs Unknown	0.84 (0.39–1.82)	0.655
	>3-6years vs <1year	0.75 (0.46–1.22)	0.248
	>6-8years vs >8-10years	1.32 (0.87–1.99)	0.19
	>6-8years vs >10-12years	0.94 (0.62–1.41)	0.749
	>6-8years vs >12years	0.77 (0.5–1.18)	0.233
	>6-8years vs Unknown	0.89 (0.41–1.94)	0.77
	>6-8years vs <1year	0.8 (0.48–1.31)	0.366
	>8-10years vs >10-12years	0.71 (0.48–1.05)	0.089
	>8-10years vs >12years	0.58 (0.39–0.88)	0.011
	>8-10years vs Unknown	0.68 (0.31–1.46)	0.318
	>8-10years vs <1year	0.6 (0.37–0.98)	0.04
	>10-12years vs >12years	0.82 (0.54–1.25)	0.357
	>10-12years vs Unknown	0.95 (0.44–2.06)	0.901
	>10-12years vs <1year	0.85 (0.52–1.38)	0.511
	>12years vs Unknown	1.16 (0.53–2.52)	0.714
	>12years vs <1year	1.03 (0.63–1.7)	0.899
	Unknown vs <1year	0.89 (0.39–2.02)	0.786
Breed group	^†^Overall	─	0.3
	Hound vs Mixed	0.86 (0.54–1.39)	0.54
	Hound vs non-Sporting	0.88 (0.5–1.55)	0.647
	Hound vs Sporting	0.97 (0.6–1.55)	0.889
	Hound vs Terrier	0.81 (0.48–1.38)	0.443
	Hound vs Toy	1.18 (0.68–2.07)	0.554
	Hound vs Unknown	0.92 (0.43–1.93)	0.816
	Hound vs Working	0.84 (0.5–1.44)	0.531
	Hound vs Herding	0.55 (0.32–0.95)	0.033
	Mixed vs non-Sporting	1.02 (0.63–1.64)	0.95
	Mixed vs Sporting	1.12 (0.78–1.61)	0.53
	Mixed vs Terrier	0.94 (0.61–1.45)	0.791
	Mixed vs Toy	1.37 (0.86–2.2)	0.184
	Mixed vs Unknown	1.06 (0.54–2.1)	0.864
	Mixed vs Working	0.98 (0.63–1.51)	0.923
	Mixed vs Herding	0.64 (0.41–1.01)	0.053
	Non-Sporting vs Sporting	1.11 (0.69–1.78)	0.682
	Non-Sporting vs Terrier	0.93 (0.55–1.58)	0.786
	Non-Sporting vs Toy	1.35 (0.77–2.38)	0.294
	Non-Sporting vs Unknown	1.05 (0.49–2.22)	0.908
	Non-Sporting vs Working	0.96 (0.56–1.65)	0.892
	Non-Sporting vs Herding	0.63 (0.36–1.09)	0.101
	Sporting vs Terrier	0.84 (0.55–1.29)	0.426
	Sporting vs Toy	1.22 (0.77–1.95)	0.393
	Sporting vs Unknown	0.95 (0.48–1.87)	0.873
	Sporting vs Working	0.87 (0.57–1.34)	0.535
	Sporting vs Herding	0.57 (0.37–0.89)	0.014
	Terrier vs Toy	1.46 (0.86–2.46)	0.158
	Terrier vs Unknown	1.13 (0.55–2.32)	0.748
	Terrier vs Working	1.04 (0.63–1.7)	0.884
	Terrier vs Herding	0.68 (0.41–1.13)	0.135
	Toy vs Unknown	0.77 (0.37–1.63)	0.497
	Toy vs Working	0.71 (0.42–1.21)	0.207
	Toy vs Herding	0.47 (0.27–0.8)	0.006
	Unknown vs Working	0.92 (0.45–1.9)	0.826
	Unknown vs Herding	0.6 (0.29–1.26)	0.177
	Working vs Herding	0.66 (0.39–1.09)	0.104
Sample source/sample type	^†^Overall	─	0.1856
	Ear & ocular vs Feces	0.81 (0.41–1.61)	0.543
	Ear & ocular vs Respiratory tract	0.47 (0.24–0.94)	0.033
	Ear & ocular vs Skin	1.02 (0.37–2.78)	0.969
	Ear & ocular vs Urine & bladder	0.79 (0.47–1.3)	0.348
	Ear & ocular vs Uterus, vagina, vulva	1.47 (0.5–4.27)	0.485
	Ear & ocular vs Wounds	0.49 (0.22–1.08)	0.075
	Ear & ocular vs All others	0.74 (0.42–1.28)	0.279
	Ear & ocular vs Abdominal cavity/fluid	1.26 (0.55–2.86)	0.587
	Feces vs Respiratory tract	0.58 (0.29–1.18)	0.132
	Feces vs Skin	1.26 (0.46–3.47)	0.649
	Feces vs Urine & bladder	0.97 (0.58–1.63)	0.917
	Feces vs Uterus, vagina, vulva	1.81 (0.62–5.32)	0.278
	Feces vs Wounds	0.6 (0.27–1.34)	0.214
	Feces vs All others	0.91 (0.52–1.61)	0.749
	Feces vs Abdominal cavity/fluid	1.56 (0.68–3.56)	0.296
	Respiratory tract vs Skin	2.18 (0.79–5.96)	0.134
	Respiratory tract vs Urine & bladder	1.67 (0.99–2.81)	0.055
	Respiratory tract vs Uterus, vagina, vulva	3.1 (1.06–9.15)	0.039
	Respiratory tract vs wounds	1.03 (0.46–2.31)	0.942
	Respiratory tract vs all others	1.56 (0.88–2.77)	0.125
	Respiratory tract vs Abdominal cavity/fluid	2.67 (1.16–6.13)	0.021
	Skin vs Urine & bladder	0.77 (0.32–1.88)	0.566
	Skin vs Uterus, vagina, vulva	1.44 (0.39–5.27)	0.586
	Skin vs Wounds	0.48 (0.16–1.42)	0.179
	Skin vs All others	0.72 (0.29–1.82)	0.488
	Skin vs Abdominal cavity/fluid	1.23 (0.41–3.71)	0.713
	Urine & bladder vs Uterus, vagina, vulva	1.87 (0.71–4.91)	0.207
	Urine & bladder vs Wounds	0.62 (0.32–1.18)	0.147
	Urine & bladder vs All others	0.94 (0.68–1.29)	0.686
	Urine& bladder vs Abdominal cavity/fluid	1.6 (0.81–3.17)	0.178
	Uterus, vagina, vulva vs Wounds	0.33 (0.11–1.04)	0.059
	Uterus, vagina, vulva vs All others	0.5 (0.19–1.36)	0.175
	Uterus, vagina, vulva vs Abdominal cavity/fluid	0.86 (0.27–2.75)	0.796
	Wounds vs All others	1.52 (0.76–3.01)	0.237
	Wounds vs Abdominal cavity/fluid	2.59 (1.03–6.49)	0.043
	All others vs Abdominal cavity/fluid	1.71 (0.83–3.51)	0.146

^†^Overall = overall effect of host factor on AMR.

#### Adjusted associations

All host factors found to be widely significantly associated (*P*≤ 0.15) with AMR in the univariable logistic regression models were included in the multivariable logistic regression analyses. Thus, for AMR, age (*P* = 0.0149), breed (*P* = 0.0007) and sample source/sample type (*P* < .0001) were included in the multivariable model. All three host factors were retained in the final multivariable model (Table 7) which showed significant associations between AMR and age (*P* = 0.009), breed (*P* = 0.0007), and sample isolation site/source (*P*<0.0001). The Hosmer and Lemeshow Goodness-of-Fit Test showed that this model best fit these data (χ^2^ = 8.05, DF = 8, *P* = 0.429). The multivariable model showed that controlling for breed and specimen source, the odds of AMR in isolates from dogs aged 1 to 3 years were 1.63 times as high as the AMR odds in isolates from dogs aged between 6 and 8 years and isolates from dogs aged greater than 10 years were more likely to be antimicrobial resistant than those isolated from other age groups. Based on the non-significant unadjusted associations (using a liberal α = 0.15), a multivariable model for the association between the host factors and MDR was not built.

**Table 7:** Multivariable binary logistic regression model of the associations between host factors and antimicrobial resistance among *Escherichia coli* isolated from samples from Indiana.

Host factors	Category	OR (95% CI)	*P* Value
Age	^†^Overall	─	0.009
	1-3years vs >3-6years	1.31 (0.9–1.9)	0.159
	1-3years vs >6-8years	1.63 (1.13–2.36)	0.009
	1-3years vs >8-10years	1.35 (0.94–1.94)	0.103
	1-3years vs >10-12years	0.89 (0.61–1.3)	0.543
	1-3years vs >12years	1.08 (0.73–1.59)	0.718
	1-3years vs Unknown	1.14 (0.59–2.2)	0.697
	1-3years vs <1year	1.5 (0.98–2.29)	0.064
	>3-6years vs >6-8years	1.25 (0.91–1.72)	0.167
	>3-6years vs >8-10years	1.04 (0.76–1.41)	0.83
	>10-12years vs >3-6years	1.47 (1.06–2.05)	0.023
	>3-6years vs >12years	0.82 (0.58–1.16)	0.264
	>3-6years vs Unknown	0.87 (0.46–1.64)	0.671
	>3-6years vs <1year	1.15 (0.78–1.69)	0.493
	>6-8years vs >8-10years	0.83 (0.61–1.12)	0.221
	>10-12years vs >6-8years	1.84 (1.33–2.55)	0.0003
	>12years vs >6-8years	1.52 (1.09–2.12)	0.014
	>6-8years vs Unknown	0.7 (0.37–1.31)	0.26
	>6-8years vs <1year	0.92 (0.62–1.35)	0.654
	>10-12years vs >8-10years	1.52 (1.11–2.1)	0.009
	>8-10years vs >12years	0.8 (0.58–1.1)	0.165
	>8-10years vs Unknown	0.84 (0.45–1.57)	0.591
	>8-10years vs <1year	1.11 (0.76–1.62)	0.598
	>10-12years vs >12years	1.21 (0.86–1.7)	0.271
	>10-12years vs Unknown	1.28 (0.68–2.42)	0.442
	>10-12years vs <1year	1.69 (1.13–2.51)	0.01
	>12years vs Unknown	1.06 (0.56–2.01)	0.858
	>12years vs <1year	1.39 (0.93–2.09)	0.11
	Unknown vs <1year	1.31 (0.68–2.55)	0.422
Breed group	^†^Overall	─	0.0007
	Hound vs Mixed	0.92 (0.64–1.31)	0.632
	Hound vs non-Sporting	0.67 (0.42–1.05)	0.081
	Hound vs Sporting	0.94 (0.66–1.35)	0.749
	Hound vs Terrier	0.47 (0.3–0.73)	0.0008
	Hound vs Toy	0.97 (0.64–1.46)	0.865
	Hound vs Unknown	1.3 (0.75–2.25)	0.343
	Hound vs Working	0.93 (0.62–1.41)	0.742
	Herding vs Hound	1.68 (1.08–2.63)	0.022
	Mixed vs non-Sporting	0.73 (0.49–1.08)	0.114
	Mixed vs Sporting	1.03 (0.78–1.36)	0.832
	Terrier vs Mixed	1.95 (1.33–2.86)	0.0006
	Mixed vs Toy	1.05 (0.74–1.49)	0.772
	Mixed vs Unknown	1.42 (0.87–2.34)	0.165
	Mixed vs Working	1.02 (0.73–1.43)	0.913
	Mixed vs Herding	0.65 (0.44–0.95)	0.027
	Non-Sporting vs Sporting	1.42 (0.96–2.1)	0.079
	Non-Sporting vs Terrier	0.71 (0.44–1.13)	0.15
	Non-Sporting vs Toy	1.45 (0.93–2.28)	0.104
	Non-Sporting vs Unknown	1.96 (1.11–3.47)	0.021
	Non-Sporting vs Working	1.4 (0.91–2.17)	0.127
	Non-Sporting vs Herding	0.89 (0.56–1.43)	0.642
	Terrier vs Sporting	2.01 (1.38–2.93)	0.0003
	Sporting vs Toy	1.02 (0.72–1.44)	0.9
	Sporting vs Unknown	1.38 (0.84–2.26)	0.2
	Sporting vs Working	0.99 (0.71–1.38)	0.949
	Sporting vs Herding	0.63 (0.43–0.92)	0.016
	Terrier vs Toy	2.06 (1.33–3.2)	0.001
	Terrier vs Unknown	2.78 (1.59–4.86)	0.0003
	Terrier vs Working	1.99 (1.3–3.06)	0.002
	Terrier vs Herding	1.27 (0.8–2.01)	0.317
	Toy vs Unknown	1.35 (0.79–2.32)	0.275
	Toy vs Working	0.97 (0.65–1.44)	0.872
	Herding vs Toy	1.62 (1.05–2.51)	0.03
	Unknown vs Working	0.72 (0.42–1.22)	0.22
	Unknown vs Herding	0.46 (0.26–0.8)	0.006
	Herding vs Working	1.57 (1.03–2.4)	0.037
Sample source/sample type	^†^Overall	─	< .0001
	Ear & ocular vs Feces	0.8 (0.45–1.43)	0.446
	Respiratory tract vs Ear & ocular	2.2 (1.12–4.34)	0.023
	Ear & ocular vs Skin	0.9 (0.37–2.18)	0.814
	Ear & ocular vs Urine & bladder	1.59 (1.06–2.39)	0.026
	Ear & ocular vs Uterus, vagina, vulva	1.91 (0.92–3.94)	0.081
	Ear & ocular vs Wounds	0.64 (0.3–1.35)	0.24
	Ear & ocular vs All others	0.97 (0.61–1.53)	0.889
	Ear & ocular vs Abdominal cavity/fluid	1.24 (0.66–2.35)	0.504
	Feces vs Respiratory tract	0.57 (0.28–1.15)	0.115
	Feces vs Skin	1.13 (0.46–2.79)	0.795
	Feces vs Urine & bladder	1.99 (1.27–3.13)	0.003
	Feces vs Uterus, vagina, vulva	2.39 (1.13–5.04)	0.022
	Feces vs Wounds	0.8 (0.37–1.73)	0.573
	Feces vs All others	1.22 (0.74–1.99)	0.44
	Feces vs Abdominal cavity/fluid	1.56 (0.8–3.04)	0.191
	Respiratory tract vs Skin	1.98 (0.75–5.2)	0.166
	Respiratory tract vs Urine & bladder	3.5 (1.99–6.13)	< .0001
	Respiratory tract vs Uterus, vagina, vulva	4.2 (1.84–9.56)	0.0006
	Respiratory tract vs wounds	1.41 (0.61–3.26)	0.425
	Respiratory tract vs all others	2.13 (1.18–3.86)	0.013
	Respiratory tract vs Abdominal cavity/fluid	2.74 (1.3–5.77)	0.008
	Skin vs Urine & bladder	1.77 (0.79–3.94)	0.165
	Skin vs Uterus, vagina, vulva	2.12 (0.78–5.77)	0.141
	Skin vs Wounds	0.71 (0.26–1.96)	0.511
	Skin vs All others	1.08 (0.47–2.46)	0.861
	Skin vs Abdominal cavity/fluid	1.38 (0.54–3.54)	0.499
	Urine & bladder vs Uterus, vagina, vulva	1.2 (0.64–2.24)	0.566
	Wounds vs Urine & bladder	2.49 (1.3–4.74)	0.006
	Urine & bladder vs All others	0.61 (0.47–0.8)	0.0003
	Urine& bladder vs Abdominal cavity/fluid	0.78 (0.47–1.32)	0.356
	Wounds vs Uterus, vagina, vulva	2.98 (1.24–7.2)	0.015
	Uterus, vagina, vulva vs All others	0.51 (0.26–0.98)	0.042
	Uterus, vagina, vulva vs Abdominal cavity/fluid	0.65 (0.3–1.44)	0.29
	Wounds vs All others	1.52 (0.77–2.98)	0.23
	Wounds vs Abdominal cavity/fluid	1.95 (0.87–4.37)	0.107
	All others vs Abdominal cavity/fluid	1.28 (0.74–2.24)	0.378

^†^Overall = overall effect of host factor on AMR.

## Discussion

In the present study, we found significant trends in susceptibility, total AMR and MDR in canine *E*. *coli* isolates, and we identified significant associations between AMR and dog age, breed, and the source of the specimens. We found significant declines in the susceptibility to cefalexin, cefazolin, and cephalothin which are 1^st^ generation cephalosporins and to cefpodoxime and ceftazidime which are 3^rd^ generation cephalosporins. Similar to our study, a previous study found high level resistance to commonly used beta lactams (penicillins, cephalosporins) in dogs in the United States [16]. Particularly, 39.7% of all the isolates in the present study were not susceptible to amoxicillin-clavulanic acid and 52.3% were not susceptible to ampicillin, and susceptibility to these drugs significantly declined over time. Similar to our findings, a previous study by Thungrat and others reported high-level resistance (45%) to amoxicillin-clavulanic acid and 52.7% to ampicillin among *E*. *coli* isolated from dogs in the United States [16]. It is important to note that amoxicillin-clavulanic acid is the most commonly prescribed antimicrobial in many veterinary practices [17–19] and ampicillin is also commonly used to treat bacterial infections in dogs [16]. Therefore, the decreasing trend in the proportion of isolates susceptible to antimicrobials in the beta lactam group in this study could be due to selection pressure from antimicrobial use. For the fluoroquinolone drugs, 21.8% of all the isolates tested were not susceptible to enrofloxacin. A previous study conducted in the northeastern US reported that nearly 20% of the *E*. *coli* isolated from dogs during the period 2004–2011 were resistant to enrofloxacin [20]. Also, among the fluoroquinolone antimicrobials, the decline in susceptibility to orbifloxacin observed could be associated with selection pressure from antimicrobial use.

The level of AMR in *E*. *coli* is a good indicator of AMR in bacterial pathogens of dogs and other species [21, 22] because of its ubiquitous nature and its ability to act as a reservoir of AMR genes that can transferred to other pathogens through horizontal gene transfer [23]. Additionally, AMR in *E*. *coli* is suggested to be a good sentinel of the effects of selective pressure from AMU [24]. Therefore, the significant increase in AMR *E*. *Coli* observed in this study could be an indicator of an increasing AMR trend among other pathogenic bacteria in the dog populations served by this diagnostic laboratory. This suggests for a need for more concerted efforts in controlling AMR in small animal practice through judicious AMU. The decreasing trend observed for MDR could have resulted from the varying susceptibility trends observed for individual antimicrobials where some individual drugs had decreasing susceptibility trends while others had increasing susceptibility. Corner and others attributed similar decreases in MDR in *Staphylococcus spp*. to variability in individual drug susceptibility [11].

The total lack of susceptibility to clindamycin and erythromycin observed is due to intrinsic resistance [9]. Enterobacteriaceae such as *E*. *coli* are known to be intrinsically resistant to lincosamides and macrolides such as clindamycin and erythromycin respectively. This information is provided here to guide veterinary clinicians who might find it useful when deciding which antimicrobial to select.

We found high susceptibility of the isolates to amikacin (97.6% susceptibility in 2010 and 97.3% in 2019) and observed a significant increase in susceptibility to this drug. Similar to our findings, a previous study that investigated the antimicrobial susceptibility patterns of *E*. *coli* in dogs and cats in the United States found only 0.7% of 2,390 canine *E*. *coli* isolates were resistant to amikacin [16]. Another study in Canada found 93.8% of 3,364 canine *E*. *coli* isolates were susceptible to Amikacin [18]. The high susceptibility and increasing trend in susceptibility to amikacin observed in the present study could be indicative of limited use of this antimicrobial in small animal practice in Indiana. The limited use of this drug could be associated with concerns about aminoglycoside toxicity. Similar to the results in amikacin, we found a near perfect susceptibility to imipenem suggesting that imipenem is rarely used in the treatment of bacterial diseases of dogs in Indiana. Imipenem belongs to the carbapenem antimicrobial class and is used in the treatment of multidrug resistant Enterobacteriaceae e.g. *E*. *coli* [25]. Perhaps this finding could reflect adherence by small animal clinicians to the guidelines for carbapenem use provided by the International Society for Companion Animal Infectious Diseases (ISCAID). The ISCAID recommends that carbapenems should be used only if the pathogen is proven to be resistant to all other reasonable antimicrobial options and susceptibility to the carbapenem chosen is documented [25].

In the present study, 61% of the *E*. *coli* isolates were found in specimens submitted from the urinary tract. This finding is similar to the findings in previous studies in the U.S. where most of the *E*. *coli* were isolated from the urinary tract [16, 20]. This suggests that urinary tract infections could have been the major reason for canine sample submission to this laboratory. However, 3.7% of the *E*. *coli* isolates were from the respiratory tract and these respiratory tract isolates were more likely to be antimicrobial resistant than those isolated from the urogenital tract (urine, bladder, uterus, vagina, and vulva), and the abdominal cavity. This is in contrast to a previous study in the north eastern United States which reported that multidrug resistance was more likely among urinary *E*. *coli* than in *E*. *coli* isolated from other canine body sites [20]. *E*. *coli* is known to be involved in respiratory tract infections in dogs and has been isolated from respiratory tract samples [26, 27]. Possibly, the higher AMR observed in the respiratory tract isolates in our study could be due to selection pressure resulting from AMU targeting respiratory tract infections in these dogs. There is a need for an in-depth study of AMR among *E*. *coli* causing respiratory disease.

In the present study, we found that *E*. *coli* isolated from dogs older than 10 years were more likely to be resistant to antimicrobials when compared to *E*. *coli* isolated from younger dogs after controlling for breed and specimen source. This finding could be due to selection pressure from prior/routine antimicrobial use in dogs in this category since dogs older than 10 years are more likely to have been treated with antimicrobials multiple times when compared to younger dogs. Previous studies found prior use of antimicrobials was a risk factor for AMR in dogs [28, 29] and AMR *E*. *coli* was common among vet-visiting dogs [30]. Specifically, prior exposure to some antimicrobials such as fluoroquinolones may select for antimicrobial resistant *E*. *coli* in dogs that could persist long after antimicrobial therapy [31, 32]. Recurrent *E*. *coli* infections are possible because *E*. *coli* possess multiple adaptations for survival and persistence in the host [33]. Dogs older than 10 years are generally considered geriatric and are likely to have weakened immune systems due to old age, and as a result, could be susceptible to frequent infections necessitating antimicrobial use. Also, selection pressure from prior AMU could be the reason why isolates from dogs aged 1 to 3 years were 1.63 times more likely to be antimicrobial resistant when compared to those from dogs between 6 and 8 years of age. From a public health standpoint, the role of dogs aged older than 10 years and those aged 1 to 3 years in the dissemination of AMR *E*. *coli* needs to be further investigated. The implications are that humans in close contact with dogs in these age groups would be at a higher risk of exposure to AMR *E*. *coli*. Veterinarians should be made aware of the potential role of dogs aged older than10 years and those aged 1 to 3 years in the spread of AMR *E*. *coli*. Generally, owners of older dogs need to be aware of the AMR *E*. *coli* risk in older dogs and should be encouraged to observe infection prevention measures such as hand washing with soap and clean water after handling their animals.

The association between AMR and breed reported in this study is surprising. We found that terriers and herding dogs were more likely to harbor AMR *E*. *coli* when compared to other breed categories. This is an interesting finding that needs to be further investigated as no previous study has elucidated this.

One limitation of this study was the lack of data related the clinical history of the dogs from which samples were collected. This prevented us from discerning the severity of the disease the dog presented with. Further, the lack of specific information regarding prior antimicrobial use in the dogs included in the study limits the inferences that can be made regarding AMU and its relationship with subsequent development of AMR.

## Conclusions

Our findings suggest that AMR in *E*. *coli* in dogs could be increasing in the state of Indiana. Dogs aged more than 10 years and those aged 1 to 3 years could play a role in the spread of AMR. *E*. *coli* in dogs in Indiana are likely to be highly susceptible to aminoglycosides (e.g., amikacin) and to carbapenems (e.g., imipenem). The findings of this study should inform efforts aimed at addressing the AMR challenge and may prove useful in guiding small animal clinicians in the state of Indiana in choosing appropriate antimicrobials for empiric therapy.

## Supporting information

S1 File (DOCX)
